# Effect of Abutment Geometry and Luting Agents on the Vertical Marginal Discrepancy of Cast Copings on Implant Abutments: An In Vitro Study

**DOI:** 10.1155/2021/9950972

**Published:** 2021-06-21

**Authors:** Jose Rosas, Frank Mayta-Tovalino, Violeta Malpartida-Carrillo, Arnaldo Munive Degregori, Roman Mendoza, Maria E. Guerrero

**Affiliations:** ^1^Oral Implantology Department, Faculty of Stomatology, Universidad Peruana Cayetano Heredia, Lima, Peru; ^2^School of Stomatology, Universidad Privada San Juan Bautista, Lima, Peru; ^3^Postgraduate Department, CHANGE Research Working Group, Faculty of Health Sciences, Universidad Científica del Sur, Lima, Peru; ^4^Academic Department of Rehabilitative Stomatology, Faculty of Dentistry, Universidad Nacional Mayor de San Marcos, Lima, Peru; ^5^Postgraduate Department, Faculty of Dentistry, Universidad Nacional Federico Villarreal, Lima, Peru; ^6^Department of Medico Surgical Stomatology, Faculty of Dentistry, Universidad Nacional Mayor de San Marcos, Lima, Peru

## Abstract

**Aim:**

Vertical marginal discrepancy (VMD) influences the success of implant-supported restorations. However, there is little literature that has investigated the influence of geometry and cementing agent on changes in VMD of metal copings on implant abutments. The objective was to evaluate the effect of the geometry of the abutment and cementing agents on VMD.

**Methods:**

Cast copings were cemented on implant abutments customized cylindrical (4, 5.5, and 7 mm) and on hexagonal implant abutments (4 mm) cemented or uncemented molded copings were placed (*n* = 4, totally 64 samples) with different luting agents. The VMD of the copings were measured in the coping-abutment interface at three reference points using a stereomicroscope. The independent Student's *t* test was used for comparison between the two different abutment walls. The post hoc statistical analysis was performed by the Tukey test.

**Results:**

There was a significant VMD increase between noncemented and cemented cast copings using different luting agents. Abutment geometry and luting agents significantly influenced the VMD (*p* ≤ 0.05). Cylindrical abutment at 7 mm in height cemented with different luting agent tested showed significantly higher VMD values than cylindrical abutments of 4 mm (*p*=0.019). Hexagonal abutments with a 4 mm height showed significantly higher VMD values than cylindrical 4 mm abutments using zinc oxide noneugenol and glass ionomer cements (*p*=0.032).

**Conclusions:**

Abutment geometry and luting agents influence the VMD of cast copings cemented on implant abutment. The higher the cylindrical abutment, the greater the VMD, and hexagonal wall abutments promote greater marginal gap.

## 1. Introduction

Implant-supported restorations have shown remarkable progress in recent years and have demonstrated high survival rates of up to 95% with well-documented functional and esthetic results [1, 2]. Classically, implant-supported restorations can be cement-retained or screw-retained. Although both methods have potential uses, the ideal option has yet to be established, and the selection depends on retrievability and esthetic factors [3].

Depending on the retention method, the success of the implant rehabilitation is not free of complications and requires dynamic equilibrium between implant osseointegration and technical and biological factors. However, based on current findings, the main complications of dental implants are produced by biological factors [4, 5]. Furthermore, according to recent studies [6], the most frequent complications are technical and biological, being significantly higher in cement-retained restorations because of disadvantages related to vertical marginal discrepancies (VMD) and excess cement extrusion on the peri-implant tissues. In addition, in most cases, residual excess cement may promote bleeding, local inflammation, and suppuration [7, 8].

Vertical marginal discrepancy is a primordial requirement for long-term success. One author recommended [9] a VMD of less than 120 *μ*m after coping cementation, as originally suggested for fixed prostheses. However, other in vitro studies [10] reported mean VMD values of 63.6 *μ*m in noncemented cast copings and 116.1 *μ*m after cast coping cementation [11]. Although there is no consensus on an acceptable maximum VMD in implant frameworks, mean values below 30 *μ*m have been difficult to achieve clinically using conventional ceramic crowns [12]. Abbo et al. [13] reported that different factors can affect implant-supported restorations. The luting agent thickness exposed to the oral cavity can be determined by the width, height, taper of the abutments, and type of luting agents. In the implant context, previous studies have evaluated the VMD using implant abutments regarding cast coping fabrication [10, 11], adhesive crown cementation [9], and crown material fabrication [14]. However, to the authors' knowledge, little evidence is available on the effect of abutment geometry and luting agents on the VMD of copings cemented over implant abutments.

Considering that control of the variables that influence the VMD and the cement line is essential, the objective of this research was to evaluate the effect of abutment geometry and luting agents on the VMD of cast copings on implant abutments.

## 2. Materials and Methods

### 2.1. Study Design and Sample Size

**Figure 1:**
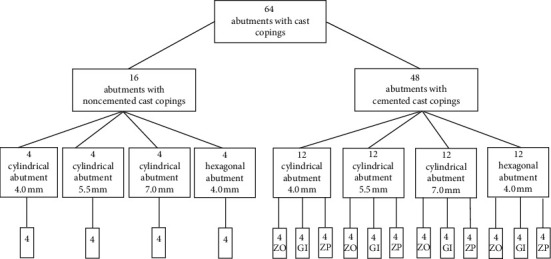
Study design. ZO: zinc oxide noneugenol; GI: glass ionomer; ZP: zinc phosphate.

**Figure 2:**
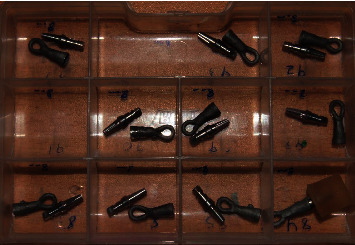
Titanium abutments.

**Figure 3:**
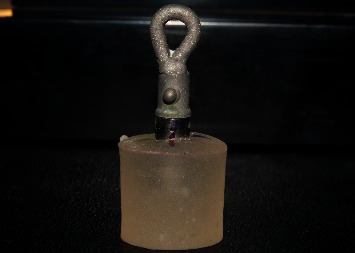
Cast coping.

This was an in vitro experimental study. The sample was calculated using the means comparison formula using Stata®15 with an alpha of 0.05 and a power of 0.8. Three types of customized cylindrical titanium abutments (12 of each) with three heights (4, 5.5, and 7 mm) and a group of 12 customized hexagonal titanium abutments of 4 mm (Titaniumfix®, São Paulo, Brazil) (Figures 1 and 2) for a total of 48 abutments received cast copings cemented with three different luting cements (4 of each). Twelve cylindrical titanium abutments with different heights (4 mm, 5.5 mm, and 7 mm) and four hexagonal titanium abutments (4 mm) only received cast copings without luting agents. First, each abutment was vertically mounted with the help of a surveyor in a block of self-cured acrylic resin (Vitacron, New Stetic, Antioquia, Colombia) measuring 1 cm in height and 1 cm in diameter (Figure 3). For cast coping fabrication, prefabricated plastic copings (Titaniumfix®, São Paulo, Brazil) were seated on the abutments and were waxed to a thickness of 1 mm in all areas. Later, the wax patterns were sprued and invested with a phosphate-bonded investment (Bellasun, Bego, Bremen, Germany).

### 2.2. In Vitro Specimen Preparation

Casting was done with Ni-Cr base-metal alloy (Wironia, Bego, Bremen, Germany) using an induction casting machine (Fornax T, Bego, Bremen, Germany). After removal of the wax sprues, the metal structures were abraded by applying 110 *μ*m aluminum oxide particles at 10 mm for 10 seconds (Basic Classic, Renfert, Germany). To identify irregularities, the inner surface of the copings was analyzed using a digital light microscope at ×40 magnification (Model T-1050, Ken-A-Vision Co, Kansas, USA). Dimensions were evaluated with a digital caliper (Mitutoyo America Corp, Aurora, IL, USA), and a custom polyvinyl siloxane matrix (Panasil®, Kettenbach, Eschenburg, Germany) was made over the coping and was used to reproduce the standard dimensions. All castings were fabricated by an experienced dental technician following the manufacturer's instructions. Casting copings and implant abutments were coded for identification during procedures. They were randomly distributed and cemented with zinc oxide without a eugenol (Rely X Temp NE, 3M ESPE, St. Paul, MN, USA), glass ionomer (Luting Cement, GC Fuji, Japan), or zinc phosphate (De Trey Zinc, Dentsply, Germany) cements.

### 2.3. Vertical Marginal Discrepancy

**Figure 4:**
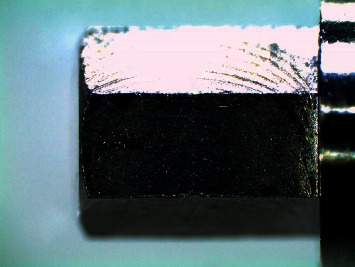
Study design.

**Figure 5:**
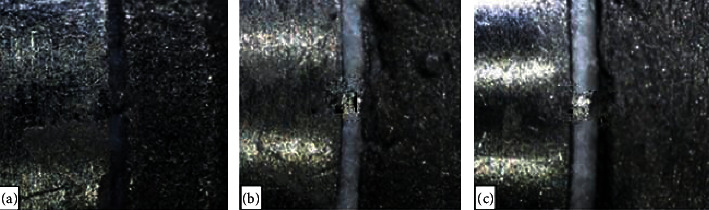
Stereomicroscope images (×200) of the coping-abutment interface after cast coping cementation. (a) Zinc oxide noneugenol (52 *μ*m). (b) Glass ionomer (46.4 *μ*m). (c) Zinc phosphate (64 *μ*m).

Subsequently, the copings were cemented on their corresponding abutments at room temperature using a thin layer of the respective luting agent assigned and considering the manufacturer's recommendations. The copings were then inserted over the abutments with 10 seconds of digital pressure and a constant axial compression load for 3 minutes at 5 kg pressure. Subsequently, the excess dental cement was removed with a curette, and the samples were stored in artificial saliva (Moi-Stir, Kingswood Laboratories, Inc., USA) for 7 days. Filamently, the copings were cleaned in ultrasound and with physiological saline (Vitasonic II, VITA, Bad Sackingen, Konstanz, Germany) for 10 min. Afterwards, the specimens were secured in a special holder and were observed using a stereomicroscope (Leica LAS EZ, Leica Microsystems, Heerbrugg, Switzerland) at ×200 magnification to evaluate VMD. The measurements were made parallel to the coping-abutment interface and photos were taken (Figures 4 and 5). Finally, the averages were considered as the marginal gap value and a computer software program (OMNIMET, Buehler, Lake Bluff, ILL, USA) was used to visually aid in quantifying the VMD space of cast copings cemented with zinc oxide noneugenol, glass ionomer, and zinc phosphate cements (Figures 5(a)–5(c)). All measurements (in micrometers) were made by the same operator.

### 2.4. Statistical Analysis

Finally, the independent Student's *t* test was used for comparison between the two different abutment walls. The post hoc statistical analysis was performed by the Tukey test using SPSS 24 (SPSS Inc., Armonk, NY, USA) software for Windows (*p* < 0.05).

## 3. Results

The mean VMD (*μ*m), standard deviation, minimum, and maximum values for each cast coping cemented or noncemented on different abutment geometries are presented in Table 1. The results showed a significant increase in VMD between noncemented cast copings compared with other cast copings cemented on cylindrical abutments of 4 mm, 5.5 mm, 7 mm, and hexagonal 4 mm abutments using the different tested luting agents (*p* < 0.05).

The bivariate test was used to determine if the VMD was influenced by the abutment geometry and the luting agent. Both variables significantly influenced the VMD (*p* < 0.05), and the values are summarized in Table 2. The data were analyzed by ANOVA and the Student's *t* test to determine whether the abutment height and abutment wall differences were significant.

The Tukey post hoc analysis showed that the 7 mm height cylindrical abutment cemented with zinc oxide noneugenol (51.21 ± 4.82 *μ*m), glass ionomer (48.06 ± 7.16 *μ*m), and zinc phosphate (58.73 ± 20.18 *μ*m) showed significantly higher VMD values than 4 mm cylindrical abutments (*p*=0.019). In addition, Student's *t* test analysis showed that hexagonal abutments with 4 mm in height showed significantly higher VMD values than 4 mm cylindrical abutments cemented with zinc oxide noneugenol and glass ionomer (*p*=0.032). Contrarily, 4 mm cylindrical abutments showed larger VMD values than when using zinc phosphate. These results are summarized in Table 3.

## 4. Discussion

To achieve successful dental implants, a constant balance between various factors is required to achieve optimal osseointegration. This can only be achieved through a constant and dynamic balance between various mechanical and biological factors. Poor vertical marginal fit of implant-cemented restorations can lead to peri-implant dissolution and inflammation of the luting agent. This could lead to the failure of the prosthetic restoration, being a major concern when the restoration is subgingival [15, 16].

In this in vitro study, VMD was evaluated considering cemented and noncemented cast copings on implant abutments with different geometries (abutment wall and abutment height). The influence of abutment geometry and luting agents (zinc oxide noneugenol, zinc phosphate, and glass ionomer) on VMD was also evaluated. The results showed a significant increase in VMD between noncemented cast copings compared with other cemented cast copings in cylindrical abutments of 4, 5.5, 7 mm, and hexagonal 4 mm abutments. The effect of different luting agents on the VMD has been reported previously in cement-retained implant-supported restorations, [9, 11, 14, 17–19] and this increase can be related to the characteristics of the luting agents such as viscosity, shape, or size filler particles. The mean VMD of cast copings cemented on cylindrical abutments using zinc oxide noneugenol, glass ionomer, and zinc phosphate (42.67 ± 5.56; 39.73 ± 5.74; 56.87 ± 13.29 *μ*m) were lower than those reported by Koç et al. [11] (116.1 ± 28.96 *μ*m). This result may be due to differences in the abutment type because these authors reduced the occlusal abutment height 2 mm using a high-speed handpiece, whereas in the present study only customized implant abutments were used. Moreover, it should be taken into account that Koç et al. [11] cemented the copings using noneugenol acrylic (temporary luting agent) which has different characteristics with respect to the luting agents used in this study. Consequently, the cementing agents used in this study could increase the marginal space between the crown and the prosthetic abutment.

In the present study, cast copings cemented with zinc phosphate showed significantly higher VMD values. These results are according to those of previous studies [20, 21] which concluded that zinc phosphate cement showed the highest film thickness compared with glass ionomer and polycarboxylate and may be attributed to its increased viscosity. Abutment geometry and luting agent statistically influenced the VMD of the evaluated cast copings. The results of the present study showed that VMD values significantly increased when the cylindrical abutment was higher (4 mm vs. 7 mm) using the different luting agents tested.

In addition, considering the abutment wall (cylindrical vs. hexagonal), the cast copings cemented on hexagonal 4 mm abutments showed the highest VMD using zinc oxide noneugenol and glass ionomer. Previous studies [22–24] showed that the removal forces of cemented copings depend on abutment geometry and luting agents. Thus, it seems that these two variables are related to technical factors and implant treatment. In the present study, the VMD was used to evaluate vertical marginal misfit according to the classification proposed by Holmes et al. [25] who defined the term VMD as a perpendicular measurement from the internal marginal surface of the cast coping to the axial wall of the crown preparation. Several studies [9, 11, 12] have reported that VMD values below 120 *μ*m are clinically acceptable. Furthermore, some authors [26, 27] have described that the average value of VMD can reach up to 200 *μ*m. Although there is no consensus on the diameter that should be considered acceptable in implant structures, recent literature has proposed a VMD of 63.6 *μ*m or less in cement-retained implant-supported restorations [10]. In our study, no sample showed VMD values greater than 120 *μ*m; therefore, all the luting agents evaluated were within normal values and could be considered clinically acceptable according to these criteria.

There is no standard technique for the measurement of VMD. The most usual methods are the impression technique, clinical examination, and direct view of the prosthetic crown [28]. Direct evaluation can be performed using stereomicroscopy, which is easy to repeat and less expensive method, although measurement points must be selected on all surfaces of the prosthetic abutment circumference [29].

Finally, in this study, VMD was assessed using implant abutments. This technique has the advantage of being noninvasive and is very useful for analyzing VMD on different sides of the prosthetic surface of the dental coping. However, the main limitation of this research was that the internal fit of the restorations was not evaluated at the time of insertion of the crowns, which can contribute to microfiltration and dissolution of dental cement. Another limitation was that all copings were made, shaped, and cemented under ideal conditions, which may not necessarily reflect real clinical activity. Therefore, studies are needed to evaluate the clinical success of prostheses installed on dental implants considering the cementation material and the geometry of the implant abutment used.

## 5. Conclusion

Abutment geometry and luting agents influence the VMD of cast copings cemented on implant abutments. Our results show that the higher the cylindrical abutment, the greater the VMD. In addition, hexagonal wall abutments promote greater marginal gaps. Therefore, clinical decisions should not only be based on the selection of the implant type since there are also variables such as the composition of the dental cement and the geometry of the prosthetic abutment that should be considered. Vertical marginal discrepancies significantly increased after cast coping cementation but were within clinically acceptable levels.

## Figures and Tables

**Table 1:** Vertical marginal discrepancy in *μ*m for cast copings cemented or noncemented over different abutment geometries.

Abutment geometry	Luting agent	Mean	SD	Min	Max	*p* ^*∗*^
Cylindrical 4.0 mm	Zinc oxide noneugenol	38.51	5.32	31.00	42.40	0.044
	Glass ionomer	33.08	5.38	25.22	42.40	
	Zinc phosphate	47.81	6.75	40.80	54.83	
	Noncemented	27.06	5.25	27.48	35.97	

Cylindrical 5.5 mm	Zinc oxide noneugenol	38.30	6.55	29.20	44.80	0.020
	Glass ionomer	38.06	4.69	35.00	44.80	
	Zinc phosphate	64.07	12.94	49.80	79.06	
	Noncemented	29.89	5.39	28.95	38.12	

Cylindrical 7.0 mm	Zinc oxide noneugenol	51.21	4.82	45.20	55.20	0.037
	Glass ionomer	48.06	7.16	40.04	55.22	
	Zinc phosphate	58.73	20.18	42.80	85.66	
	Noncemented	28.49	5.12	30.57	40.18	

Hexagonal 4.0 mm	Zinc oxide noneugenol	42.98	8.15	31.80	50.00	0.030
	Glass ionomer	47.70	9.66	34.41	50.00	
	Zinc phosphate	43.75	5.83	38.82	51.40	
	Noncemented	29.12	5.67	29.16	40.62	

SD: standard deviation. Student's *t* test. ^*∗*^Statistically significant differences (*p* < 0.05).

**Table 2:** Vertical marginal discrepancy in *μ*m for cast copings influenced by abutment geometry and luting agent.

Abutment geometry and luting agent	Vertical marginal discrepancy		
	Mean	SD	*p*
*Abutment geometry*			
Cylindrical 4.0 mm	31.80	6.03	0.012^*∗*^
Cylindrical 5.5 mm	46.81	8.06	
Cylindrical 7.0 mm	52.67	10.72	
Hexagonal 4.0 mm	44.81	7.88	
Total	—	—	

*Luting agent*			
Zinc oxide noneugenol	42.75	6.21	0.016^*∗*^
Glass ionomer	41.72	6.72	
Zinc phosphate	53.59	11.42	
Total	—	—	

SD: standard deviation. ANOVA test. ^*∗*^Statistically significant differences (*p* < 0.05).

**Table 3:** Mean vertical marginal discrepancy in *μ*m depending on abutment geometry after cementation using different luting agents.

Abutment geometry	Luting agent			
	Mean VMD, zinc oxide noneugenol	Mean VMD, glass ionomer	Mean VMD, zinc phosphate	*p*
*Abutment height*				
Cylindrical 4.0 mm	38.51 ± 5.32^*∗*^	33.08 ± 5.38^*∗*^	47.81 ± 6.75^*∗*^	0.019
Cylindrical 5.5 mm	38.30 ± 6.55	38.06 ± 4.69	64.07 ± 12.94	
Cylindrical 7.0 mm	51.21 ± 4.82^†^	48.06 ± 7.16^†^	58.73 ± 20.18^†^	
Total	42.67 ± 5.56	39.73 ± 5.74	56.87 ± 13.29	

*Abutment wall*				
Cylindrical 4.0 mm	38.51 ± 5.32^‡^	33.08 ± 5.38^‡^	47.81 ± 6.75^‡^	0.032
Hexagonal 4.0 mm	42.98 ± 8.15^§^	47.70 ± 9.66^§^	43.75 ± 5.83^§^	
Total	40.74 ± 6.73	40.39 ± 7.52	45.78 ± 6.29	

VMD: vertical marginal discrepancy. ^*∗*^, ^†^Two-way ANOVA test, post hoc Tukey test. ^‡^, ^**§**^Student's *t* test. ^*∗*^, ^†^, ^‡^, ^§^Different superscript letters in each column indicate statistically significant differences (*p* < 0.05).

## Data Availability

The data used in this study will be available upon authorization of the corresponding author.
